# Detection of the Heterogeneous *O*-Glycosylation Profile of MT1-MMP Expressed in Cancer Cells by a Simple MALDI-MS Method

**DOI:** 10.1371/journal.pone.0043751

**Published:** 2012-08-22

**Authors:** Takuya Shuo, Naohiko Koshikawa, Daisuke Hoshino, Tomoko Minegishi, Hiroko Ao-Kondo, Masaaki Oyama, Sadanori Sekiya, Shinichi Iwamoto, Koichi Tanaka, Motoharu Seiki

**Affiliations:** 1 Division of Cancer Cell Research, Institute of Medical Science, University of Tokyo, Minato-ku, Tokyo, Japan; 2 Medical Proteomics Laboratory, Institute of Medical Science, University of Tokyo, Minato-ku, Tokyo, Japan; 3 Koichi Tanaka Mass Spectrometry Research Laboratory, Shimadzu Corporation, Nakagyo-ku, Kyoto, Japan; University of Patras, Greece

## Abstract

**Background:**

Glycosylation is an important and universal post-translational modification for many proteins, and regulates protein functions. However, simple and rapid methods to analyze glycans on individual proteins have not been available until recently.

**Methods/Principal Findings:**

A new technique to analyze glycopeptides in a highly sensitive manner by matrix-assisted laser desorption/ionization mass spectrometry (MALDI-MS) using the liquid matrix 3AQ/CHCA was developed recently and we optimized this technique to analyze a small amount of transmembrane protein separated by SDS-PAGE. We used the MALDI-MS method to evaluate glycosylation status of membrane-type 1 matrix metalloproteinase (MT1-MMP). *O*-glycosylation of MT1-MMP is reported to modulate its protease activity and thereby to affect cancer cell invasion. MT1-MMP expressed in human fibrosarcoma HT1080 cells was immunoprecipitated and resolved by SDS-PAGE. After in-gel tryptic digestion of the protein, a single droplet of the digest was applied directly to the liquid matrix on a MALDI target plate. Concentration of hydrophilic glycopeptides within the central area occurred due to gradual evaporation of the sample solution, whereas nonglycosylated hydrophobic peptides remained at the periphery. This specific separation and concentration of the glycopeptides enabled comprehensive analysis of the MT1-MMP *O*-glycosylation.

**Conclusions/Significance:**

We demonstrate, for the first time, heterogeneous *O*-glycosylation profile of a protein by a whole protein analysis using MALDI-MS. Since cancer cells are reported to have altered glycosylation of proteins, this easy-to-use method for glycopeptide analysis opens up the possibility to identify specific glycosylation patterns of proteins that can be used as new biomarkers for malignant tumors.

## Introduction

Glycosylation is a common and highly diverse co- and post-translational protein modification [1]. Accumulating evidence indicates that addition or modification of one or more sugar moieties can have diverse effects on protein function and such modifications have been implicated in different types of disease [2]. In particular, specific changes in protein *O*-glycosylation have been reported in malignant tumors [3], [4], [5]. *O*-glycosylation is initiated by the addition of an *N*-acetylgalactosamine (GalNAc) to a hydroxyl group on a serine or threonine residue followed by the subsequent addition of galactose or *N*-acetylglucosamine (GlcNAc), which forms the core structure of the glycan chain [6]. Core *O*-glycans can be further extended by other carbohydrate moieties such as fucose, and/or sialic acid. It has been reported that the core structures of *O*-glycans are involved in the metastatic capacity of cancer cells. For example, Iwai *et al*. showed that forced expression of β1,3-*N*-acetylglucosaminyltransferase 6, which adds β1,3-linked GlcNAc to GalNAc at the reducing terminus, in human fibrosarcoma FP-10 cells (a highly invasive variant of HT1080 cells) resulted in significant reduction in *in vitro* migration ability and in the formation of lung metastases *in vivo* in mice [7].

MT1-MMP is a type I transmembrane proteinase that plays crucial roles in tumor cell invasion, due to its ability to cleave a broad spectrum of extracellular matrix macromolecules including collagens and laminins, and to activate proMMP-2 [8]. MT1-MMP has a multi-domain structure with a catalytic domain (Thr^112^-Gly^285^), a hinge domain (Glu^286^-Ile^318^), a hemopexin-like domain (Cys^319^-Cys^508^) and a stem domain (Pro^509^-Ala^541^) in the extracellular region [9]. Recent studies indicate that MT1-MMP is post-translationally modified by *O*-glycan at multiple sites within the hinge domain. This modification has been implicated in substrate recognition [10], protein stability [11], and turnover of the enzyme [12]. For example, Wu *et al*. showed that MT1-MMP *O*-glycosylation affects proMMP-2 activation on the cell surface [10]. Possible glycosylation sites of MT1-MMP were suggested by amino acid sequence analysis and site-directed mutagenesis [10], [13] and the glycan moieties were analyzed using lectins and glycosidases [10], [11]. MT1-MMP is usually expressed at low levels (1–2×10^5^ molecules/cell) even in cancer cells [14]. Therefore, these biochemical methods are not suited to analyze the heterogeneous glycosylation status of MT1-MMP, particularly in clinical samples.

MALDI-MS [15], [16] is an indispensable analytical tool for elucidating both the peptide and glycan moieties of glycopeptides [17]. However, glycopeptides are generally more inefficiently ionized than nonglycosylated peptides, and furthermore, the amount of glycopeptide generated by protease digestion of a protein sample is usually quite small compared to that of nonglycosylated peptide. Therefore, prior separation of glycosylated and nonglycosylated peptides is inevitably required for MS analysis [18]. A recently developed MALDI technique using a liquid matrix 3AQ/CHCA, which is composed of 3-aminoquinoline and α-cyano-4-hydroxycinnamic acid, enabled on-target separation of glycosylated and nonglycosylated peptides based on differences in their hydrophilicity after a droplet of protease digest was applied onto the liquid matrix on a MALDI target plate [19]. This on-target separation method was employed to analyze *N*-glycosylation of ribonuclease B, which is used as a model glycoprotein. Here we applied and optimized the new MALDI-MS method to analyze the *O*-glycosylation profile of MT1-MMP expressed in cancer cells at a low level and tried to detect the heterogeneous modification status.

## Results

### On-target Separation of Peptides on a Liquid Matrix 3AQ/CHCA

**Figure 1:**
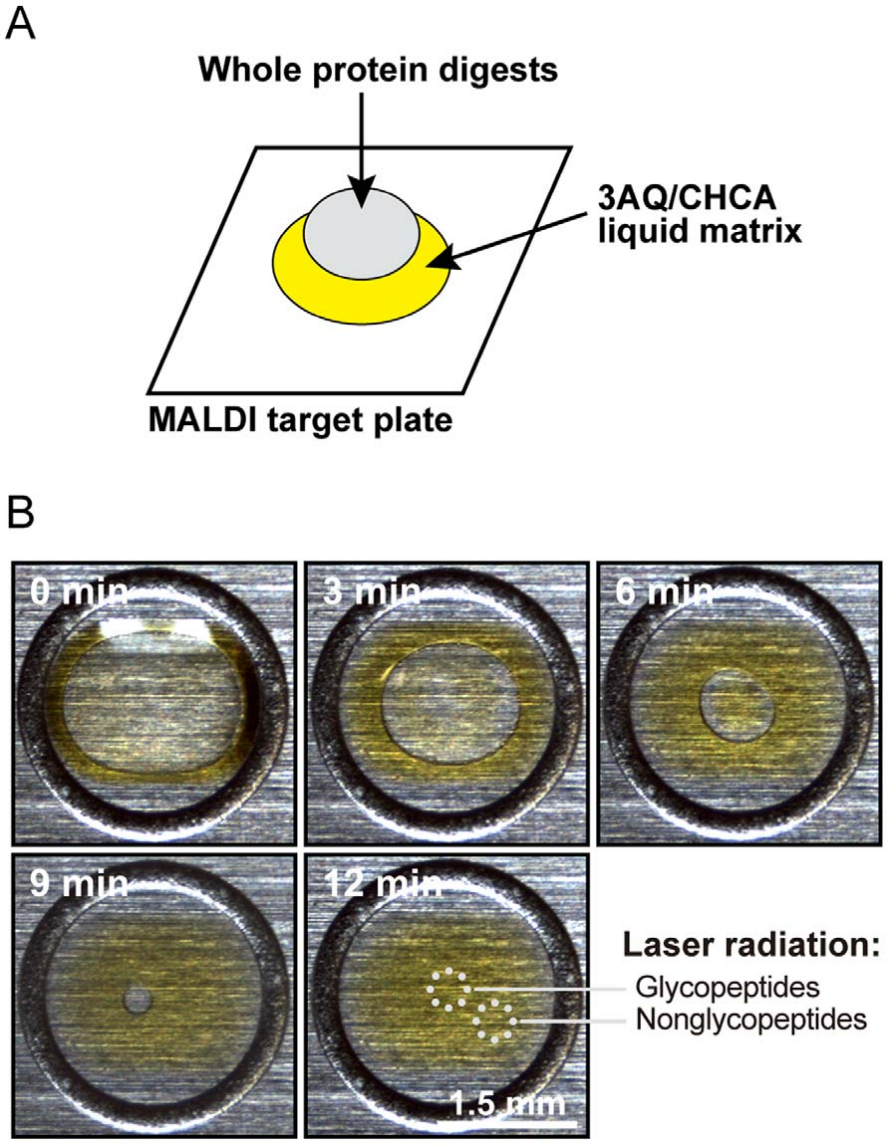
Focusing of hydrophilic peptides by the liquid matrix, 3AQ/CHCA. (A) Schematic representation of the on-target separation method described in this study. 3AQ/CHCA is first spotted onto the MALDI target plate, and then the proteolytic whole protein digests containing both glycosylated and nonglycosylated peptides are applied to the top surface of the liquid matrix. (B) Time course monitoring of the sample droplet on the liquid matrix. Evaporation of the sample solution allows the droplet to shrink such that the hydrophilic constituents are gradually concentrated in a focused droplet.

Peptides of different hydrophilicity can be separated on the liquid matrix 3AQ/CHCA spotted onto a MALDI target plate [19]. The 3AQ/CHCA matrix forms a yellow-colored viscous spot when it is applied to the target plate as illustrated in Fig. 1A. A single droplet of proteolytic digest of a protein sample containing both glycosylated and nonglycosylated peptides is then spotted onto the surface of the liquid matrix. The sample spreads uniformly on the liquid matrix and the sample solution gradually evaporates within approximately 12 min (Fig. 1B). During evaporation, hydrophilic constituents such as glycopeptides are concentrated within the central area. In contrast, hydrophobic peptides tend to be excluded from the evaporating solution and are left at the periphery of the liquid matrix.

### Detection of Glycosylated Peptides of Endogenous MT1-MMP

**Figure 2:**
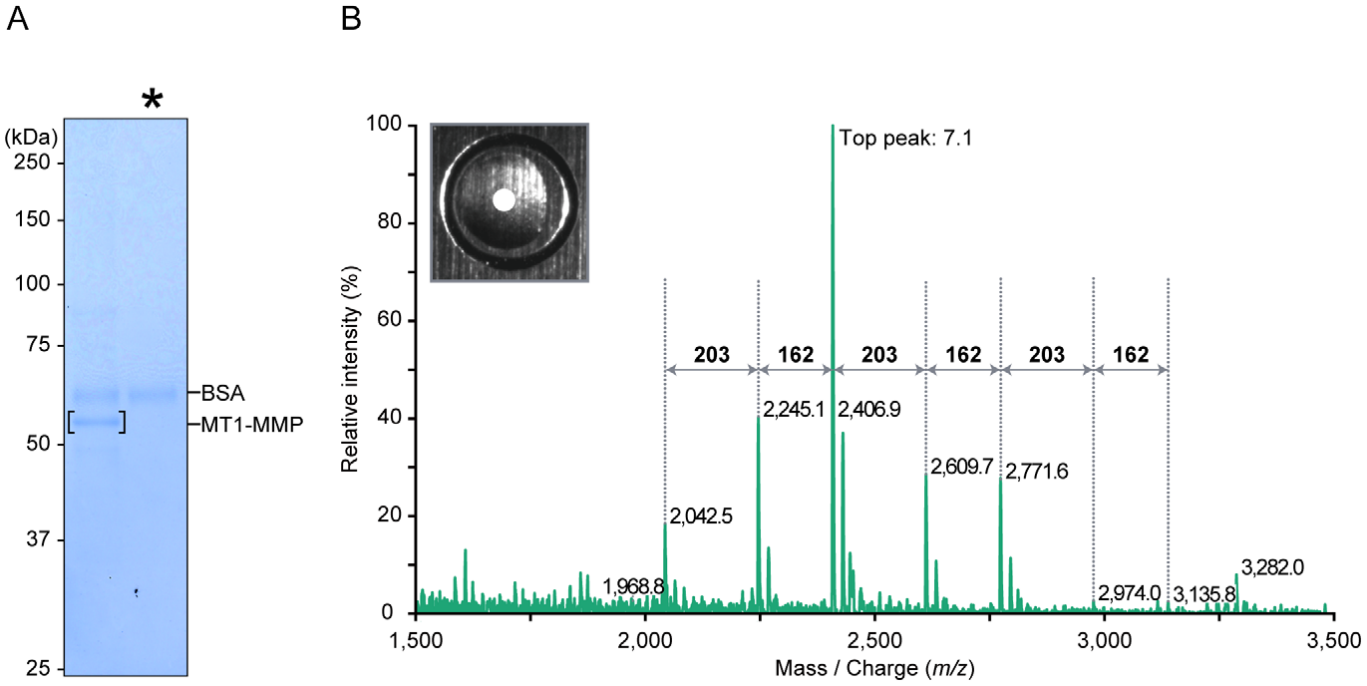
MS spectrum of tryptic MT1-MMP digests within the central area of 3AQ/CHCA. (A) Endogenous MT1-MMP was immunoprecipitated with anti-MT1-MMP antibody-conjugated resin from membrane lysates of wild-type HT1080 cells and was further separated by SDS-PAGE and staining with Coomassie Blue. Sialidase, which contains BSA as a carrier protein, was loaded in the light lane (denoted by asterisk). Numbers on the left of the panel represents molecular masses in kilodaltons (kDa). (B) The polypeptide band corresponding to MT1-MMP was excised, digested in-gel with trypsin. An aliquot of tryptic MT1-MMP digest derived from HT1080 cells was applied directly onto the liquid matrix 3AQ/CHCA on the MALDI target plate. MS spectrum was obtained within the central area of the liquid matrix (open circle [○]). A stereoscopic microscope image of the sample spot is shown in the left upper insert. Number represents the cumulative intensity of the top peak (arbitrary units).

MT1-MMP is an integral membrane proteinase expressed on the surface of aggressive cancer cells. We first tried to detect the MS spectrum generated from glycopeptides of MT1-MMP expressed in cancer cells. Endogenous MT1-MMP was immunoprecipitated from membrane lysates of human fibrosarcoma HT1080 cells using a monoclonal anti-MT1-MMP antibody prepared in our laboratory as described in *Materials and Methods*. Sialic acids, which resist ionization in the MS analysis [20], were removed using sialidase. Samples were then separated by SDS-PAGE (Fig. 2A). Dilutions of bovine serum albumin (BSA) were also applied to the gel to estimate protein amount (data not shown). The gel was stained with Coomassie Blue and approximately 150 ng of the MT1-MMP protein was excised (Fig. 2A, the band is bracketed). The isolated gel fragment was subjected to in-gel digestion with trypsin and one-sixtieth of the digest was applied directly onto the 3AQ/CHCA matrix on a MALDI target plate.

The tryptic MT1-MMP digest applied to the liquid matrix on a MALDI target plate was allowed to evaporate and the central portion of the sample load area was irradiated using a laser beam (Fig. 2B, inserted picture). The MS spectrum generated comprised several peaks (*m/z* 2,042.5, 2,245.1, 2,406.9, 2,609.7, 2,771.6, 2,974.0 and 3,135.8) and the distances between the peaks corresponded precisely to the masses of typical monosaccharides: 162 and 203 Da for hexose (Hex) and *N*-acetylhexosamine (HexNAc), respectively (Fig. 2B). Therefore, these peaks were thought to be derived from a single peptide glycosylated differentially.

### Analysis of Glycosylated Peptides

To further investigate the glycopeptide peaks derived from MT1-MMP by second and third stages of MS analyses (MS^2^ and MS^3^), we used a FLAG-tagged MT1-MMP (MT1-FLAG) expressed in HT1080 cells because of the reason that a large quantity of the FLAG-tagged protein can be purified easily and efficiently. To reproduce the native glycosylation pattern of MT1-MMP in the cells, MT1-FLAG was expressed stably at a level comparable to that of the endogenous protein in wild-type cells. We expressed MT1-FLAG in an HT1080 derivative in which the expression of endogenous MT1-MMP was knocked down using shRNA. Levels of MT1-MMP expression in the cells were confirmed by Western blot analysis (Fig. S1A). MT1-FLAG was purified from membrane lysates of the revertant cells using anti-FLAG antibody. Approximately 400 ng of the MT1-FLAG protein was resolved by SDS-PAGE (Fig. S1B) and then analyzed by the on-target separation method through MALDI-MS.

**Figure 3:**
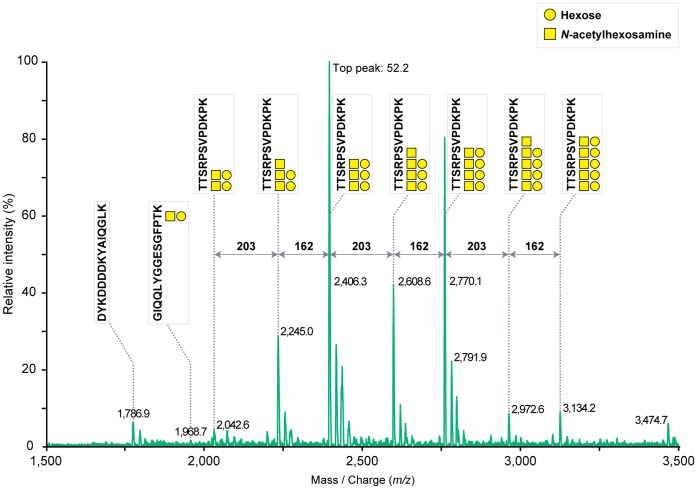
MS spectrum generated from glycopeptides of MT1-MMP. An aliquot of tryptic MT1-FLAG digest was applied directly onto the liquid matrix 3AQ/CHCA. MS spectrum was obtained within the central area of the liquid matrix. The glycan moieties and the amino acid sequences of peptides including glycosylation sites of these glycopeptides were assigned by MS^2^ and MS^3^. Number represents the cumulative intensity of the top peak (arbitrary units).

**Figure 4:**
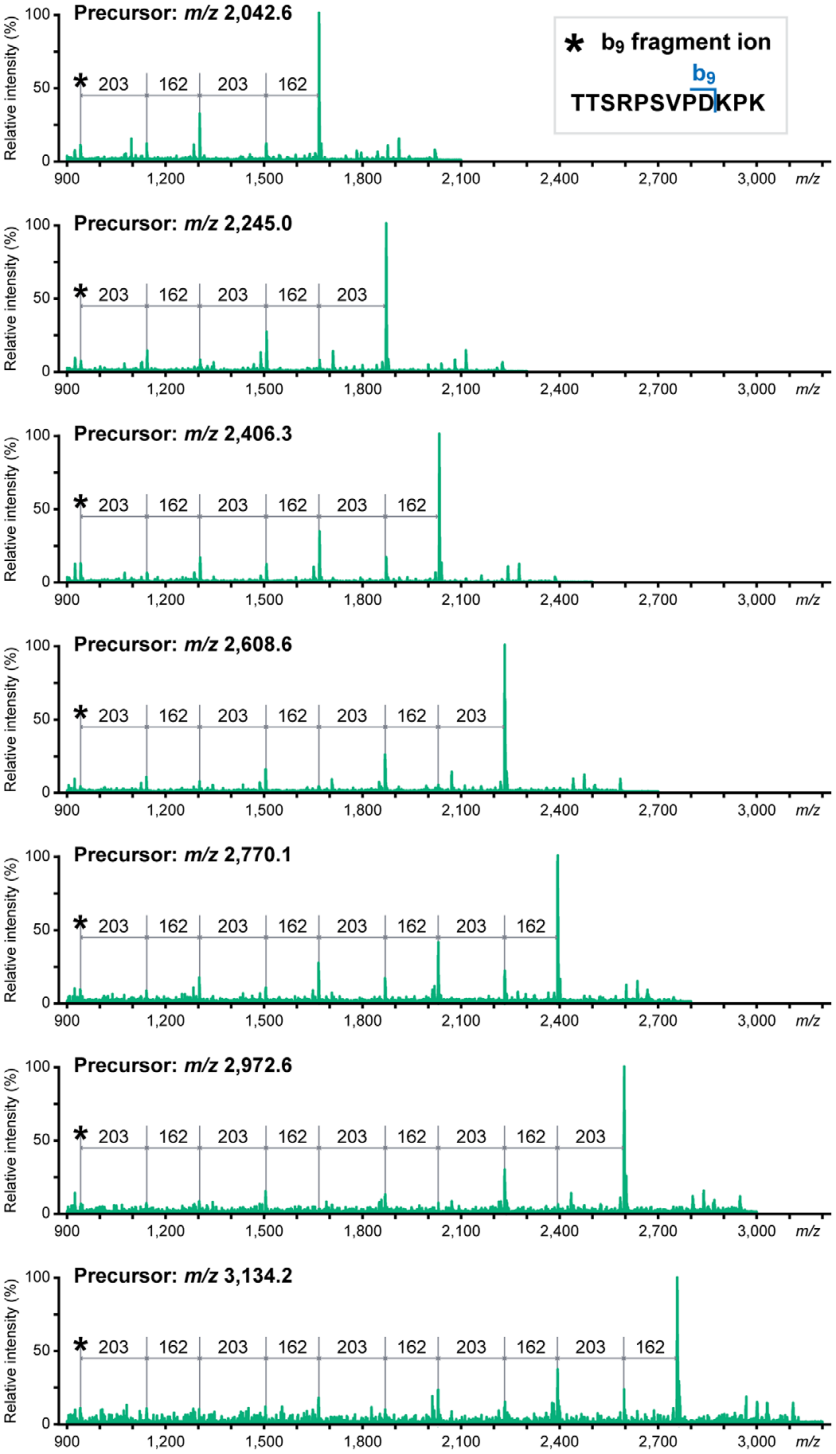
MS^2^ spectra of the ions at *m/z* 2,042.6, 2,245.0, 2,406.3, 2,608.6, 2,770.1, 2,972.6 and 3,134.2. Ion peaks derived from the MS spectrum of tryptic MT1-FLAG digests at *m/z* 2,042.6, 2,245.0, 2,406.3, 2,608.6, 2,770.1, 2,972.6 and 3,134.2 (Fig. 3) were subjected to MS^2^ analysis. These fragment ions were assigned to the loss of single monosaccharides (162 and 203 Da for Hex and HexNAc, respectively) from the same peptide ion (^299^TTSRPSVPD^307^).

**Figure 5:**
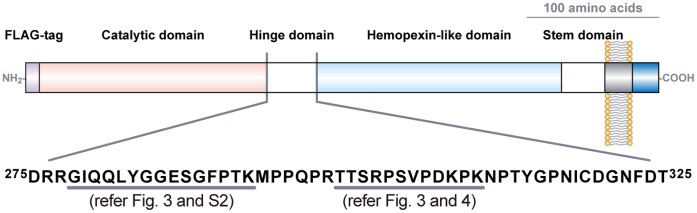
*O*-glycosylation sites of MT1-MMP. MT1-MMP is a type I transmembrane proteinase, and has a particular multidomain structure with a catalytic domain, a hinge domain, a hemopexin-like domain and a stem domain in the extracellular region. Underlines indicate glycopeptides identified in this study.

The obtained MS spectrum of MT1-FLAG was almost identical to that of the endogenous MT1-MMP (Fig. 3 versus Fig. 2B), except a hydrophilic FLAG-tag (DYKDDDDK)-containing peptide peak at *m/z* 1,786.9 in MT1-FLAG. The collision-induced dissociation of major protonated ion [M+H]^+^ peaks (*m/z* 2,042.6, 2,245.0, 2,406.3, 2,608.6, 2,770.1, 2,972.6 and 3,134.2) via MS^2^ indicated that the fragment ions correspond to a series of losses of monosaccharides from the glycosylated peptide ^299^TTSRPSVPDKPK^310^ (Fig. 4). The glycoforms of this peptide contained 2 to 5 Hex and HexNAc. Fragment ion spectra were also obtained from another glycopeptide ^278^GIQQLYGGESGFPTK^292^ as a sodiated ion [M+Na]^+^ peak at *m/z* 1,968.7 (Fig. S2A). The MS^2^ product ion at *m/z* 1,024.0 was shown to be a Hex−HexNAc containing peptide ^287^SGFPTK^292^ by MS^3^ fragmentation (Fig. S2B). A summary of the identified glycopeptides are indicated schematically on the structure of MT1-MMP (Fig. 5). Thus, we have been able to detect glycosylated peptides of MT1-MMP by MS analysis of a whole protein extract from a gel. Such glycopeptide peaks were not obtained when the same sample was analyzed using a conventional solid matrix DHB [21] as presented in Fig. S3. These results clearly indicate the superiority of the new method using the liquid matrix 3AQ/CHCA.

### Heterogeneous Glycosylation Profile of MT1-MMP

In order to prepare a standard protein sample to set up assay conditions for glycopeptide analysis, we prepared a soluble MT1-MMP, which lacks the transmembrane domain that was expressed in Madin-Darby canine kidney (MDCK) cells. Result of the MS analysis of the soluble MT1-MMP is presented in Fig. S4. Only one major glycopeptide peak was detected for the peptide ^299^TTSRPSVPDKPK^310^. On the other hand, multiple glycopeptide peaks were detected from the same peptide of MT1-FLAG expressed in HT1080 cells (Fig. 3). Therefore, the glycosylation pattern of MT1-FLAG obtained from HT1080 cells represents a heterogeneous glycosylation profile of the peptide and it is not because of degradation or modification of the carbohydrate moieties during sample preparation and its application to the MS analysis. Taken together, this on-target separation method may become a powerful easy-to-use tool to analyze heterogeneous glycosylation patterns of proteins of choice.

### Detection of Nonglycosylated Peptides on the Same Target Plate

MS spectra of glycopeptides were preferentially obtained from the central area of the sample-loaded matrix. This result indicates that we accomplished separation of glycosylated and nonglycosylated peptides on the liquid matrix. To confirm further the separation of the nonglycosylated peptides on the matrix, we analyzed the periphery of the sample-loaded area on the liquid matrix by laser radiation. Indeed, many unmodified peptides derived from the MT1-MMP digest were detected (Fig. S5, peaks marked by stars), whereas glycopeptides were not detected within this area.

Overall, we achieved approximately 50% sequence coverage of the extracellular region of MT1-MMP using this method (Fig. S6). Thus, a whole protein analysis of glycosylated and nonglycosylated peptides was accomplished following conventional SDS-PAGE, in-gel digestion, and direct application of a single droplet of a sample digest onto the liquid matrix 3AQ/CHCA on the MALDI target plate.

### Lower Limit of Protein Amount for the Assay

**Figure 6:**
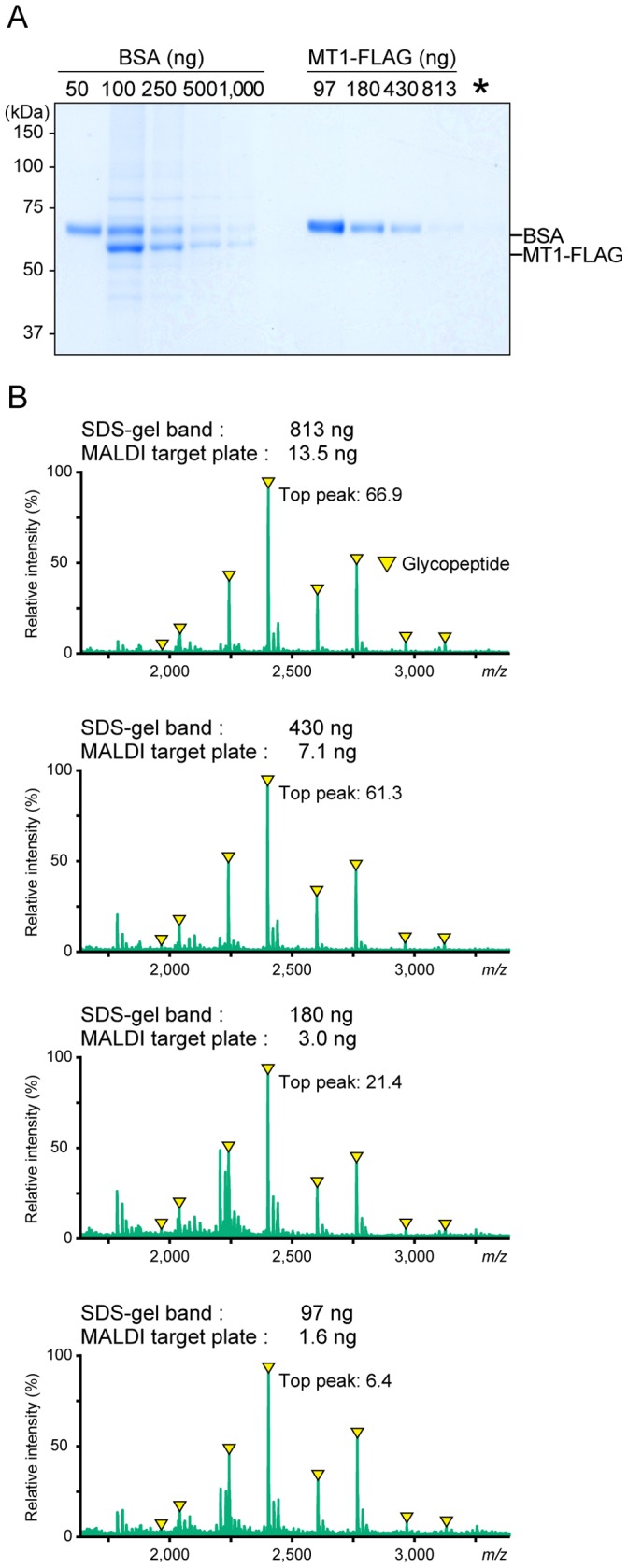
Comparison of MS spectra of tryptic MT1-MMP digests containing different amounts of MT1-MMP. (A) MT1-FLAG was serially diluted and then separated by SDS-PAGE and stained with Coomassie Blue. Sialidase, which contains BSA as a carrier protein, was loaded in the far-left lane (denoted by asterisk). Numbers on the left of the panels represent molecular masses in kilodaltons (kDa). (B) The polypeptide bands corresponding to MT1-FLAG were excised, in-gel digested with trypsin, and analyzed by MS using the liquid matrix 3AQ/CHCA. Glycopeptides contained in the whole protein digest obtained from MT1-FLAG (approximately 1.6 ng based on comparison with a BSA standard) were detected in the center of the liquid matrix. Numbers represent the cumulative intensity of the top peaks (arbitrary units). Arrowheads indicate glycopeptide ions (refer Fig. 3).

We finally determined the MS spectra generated from glycopeptides within a smaller amount of the sample protein. Different amounts of MT1-FLAG protein were subjected to SDS-PAGE and the gel was stained with Coomassie Blue. The amount of the MT1-FLAG was estimated using BSA as a standard protein (Fig. 6A). Portions of the gel containing the sample proteins were excised and analyzed similarly (Fig. 6B). Although the peak intensities of MS spectra decreased according to the sample amount, we could detect glycopeptide peaks even from the smallest amount of the sample and the amount of protein loaded onto the liquid matrix in this case was estimated to be approximately 1.6 ng.

## Discussion

Many biochemical tools are available to analyze *N*-glycosylation of proteins, whereas those for *O*-glycosylation are limited. Therefore, analysis of the *O*-glycosylation status of proteins using small amounts of sample has been technically challenging. An on-target separation method, making use of the liquid matrix 3AQ/CHCA for MALDI-MS, was developed recently and applied to analyze *N-*glycosylation of ribonuclease B. We applied this method to analyze *O*-glycosylation of MT1-MMP expressed at low levels in cancer cells.

The on-target separation method using the liquid matrix is extremely easy and rapid to analyze a mixture of glycosylated and nonglycosylated peptides compared to conventional MS analysis using DHB. Since glycosylated peptides are weakly ionized compared to nonglycosylated ones, glycopeptide signals could not be detected when the same MT1-FLAG digest was applied to DHB matrix (Fig. S3). Therefore, DHB cannot be used for glycopeptide analysis without prior processing for separation of the latter. In contrast, prior separation of glycosylated and nonglycosylated peptides is not needed for MS analysis when using 3AQ/CHCA. Since an additional separation step following proteolytic digestion is not needed, only a small amount of protein sample was sufficient to obtain clear results as demonstrated in Fig. 6. Furthermore, the liquid matrix 3AQ/CHCA is compatible with not only analyses of Coomassie blue-stained protein samples but also that of silver-stained ones (data not shown). Silver staining allows detection of most proteins since it is more sensitive than staining with Coomassie blue. Taken together, the MALDI-MS method using 3AQ/CHCA may make it possible to investigate the glycosylation of a protein that is expressed in minute amounts and/or present in specific surface area such as lipid rafts where the purification is more complex.

In this study, we mainly used MT1-FLAG protein expressed in HT1080 cells to evaluate the versatility of the new application of the liquid matrix to analyze glycoproteins by MS. This is simply because we have been studying MT1-MMP expressed in cancer cells and wanted to use it as a standard transmembrane protein expressed at low levels. We detected glycosylated MT1-MMP peptide peaks derived from ^278^GIQQLYGGESGFPTK^292^ and ^299^TTSRPSVPDKPK^310^ (Fig. 3). Therefore, the Thr and Ser residues within these peptides represent possible *O*-glycosylation sites. Thr^291^, Thr^299^, Thr^300^, and Ser^301^ were suggested to be sites of *O*-glycosylation by mutation analysis [10], [13]. In addition, we found that Ser^304^ is also glycosylated in HT1080 cells by detecting a glycopeptide signal from ^303^PSVPDKPK^310^ by MS^2^ analysis of the sodiated ion [M+Na]^+^ peak at *m/z* 2,791.9 as in Fig. 3 (data not shown). Although these results are largely confirmatory, the analysis also revealed the heterogeneous *O*-glycosylated peptide peaks from ^299^TTSRPSVPDKPK^310^ (Fig. 4). Thus, a whole protein MS analysis using liquid matrix is a powerful tool to analyze the heterogeneity of glycosylation. The method is easy-to-use even for clinical samples where specific antibodies are available for immunoaffinity purification of target proteins from cells, tissue, sera, and urine. Since altered glycosylation of proteins has been linked to cancer progression [3], [4], [5], the on-target separation method using 3AQ/CHCA will accelerate analysis of heterogeneity of *O*-glycosylation for different cancer-related proteins. If there are cancer-specific variations, the identified *O*-glycans may represent new candidate biomarkers for malignant tumors. In addition, there is increasing interest in biotechnology-based pharmaceuticals that are manufactured using living cells. The glycosylation status of these biological products has been shown to be important to its pharmacological properties [22]. The present MALDI-MS method may become a valuable tool for glycoanalysis of clinical samples as well as assessment for the quality of biological drug products.

## Materials and Methods

### Cell Culture

Human HT1080 fibrosarcoma cells (American Type Culture Collection, Manassas, VA) were maintained in Dulbecco’s modified Eagle’s medium (Invitrogen, Carlsbad, CA) supplemented with 10% fetal bovine serum, 10 µM MMI270 (a synthetic hydroxamic matrix metalloproteinase inhibitor, a kind gift of Novartis Pharma AG, Basel, Switzerland), and 1% penicillin/streptomycin at 37°C in a 5% CO_2_ incubator.

### Lentiviral Vectors for MT1-MMP Expression and Knockdown

The shRNA sequence used for knockdown of MT1-MMP was 5′-CACCGCAGCCTCTCACTACTCTTTCCGAAGAAAGAGTAGTGAGAGGCTGC-3′. A DNA fragment encoding the sequence was subcloned into pENTR/U6 TOPO (Invitrogen) and then transferred via recombination into the lentivirus vector pLenti6 BLOCK it (Invitrogen). A construct encoding human MT1-MMP tagged with the FLAG epitope (DYKDDDDK) was described previously [23]. A cDNA clone of FLAG-tagged MT1-MMP (MT1-FLAG) was amplified by the PCR using the following primers: 5′-CACCATGTCTCCCGCCCCAAGACC-3′ and 5′-TCAGACCTTGTCCAGCAGGG-3′. The amplified MT1-FLAG sequence was subcloned into pENTR/D-TOPO and then transferred via recombination into the lentiviral pLenti6/V5-DEST expression vector (Invitrogen). These lentiviral vectors were generated and used according to the manufacturer’s instructions.

### MT1-MMP Knockdown and Revertant Cell Line

MT1-FLAG was expressed in lentivirus-infected HT1080 cells (revertant cells) in which the expression of endogenous MT1-MMP was first depleted by expressing the shRNA targeting the endogenous MT1-MMP (knockdown cells). MT1-FLAG was expressed at a level similar to that of the wild-type protein based on Western blot analysis.

### Whole Cell Lysate Preparation

35 cm^2^ cell-culture dishes containing subconfluent wild-type, MT1-MMP knockdown and MT1-FLAG revertant HT1080 cells supplemented with 10 µM MMI270 were washed with PBS and lysed by boiling in 200 µL Laemmli’s sample buffer [24] containing 5% β-mercaptoethanol. A 10 µL aliquot of the lysate was used for Western blot analysis.

### Membrane Lysate Preparation

All procedures were carried out at 4°C. Sixteen 150 cm^2^ cell-culture dishes containing subconfluent cultures of wild-type or MT1-FLAG revertant HT1080 cells supplementated with 10 µM MMI270 in the media were washed with PBS and scraped in 32 mL of sucrose/Hepes buffer (0.25 M sucrose/20 mM Hepes-NaOH, pH 7.2) containing protease inhibitors (protease inhibitor cocktail set I, Calbiochem, San Diego, CA). The cells in the buffer were dounce-homogenized (10 times). The homogenate was centrifuged at 1,000×g for 7 min. The pellet was rehomogenized in 16 mL of sucrose/Hepes buffer, and the suspension was centrifuged 1,000×g for 7 min. The supernatants were combined and centrifuged at 2,000×g for 30 min. Supernatant from this centrifugation was ultracentrifuged at 105,000×g for 1 h. Pellets were resuspended in 1 mL of lysis buffer (1% Triton X−100/20 mM Hepes-NaOH, pH 7.2) containing protease inhibitors and then centrifuged at 20,000×g for 30 min at 4°C. The supernatant is referred to as the membrane lysate, resulting in a protein concentration of about 10 mg/mL. The protein concentration in the lysate was determined using a Bio-Rad Protein Assay Kit (Bio-Rad Labs, Hercules, CA) with BSA as a reference standard.

### Endogenous MT1-MMP Isolation

A monoclonal antibody for endogenous MT1-MMP isolation was prepared in our laboratory by a genetic immunization method [25] using a full-length human MT1-MMP expression plasmid. This antibody recognizes a region within a stem domain of MT1-MMP (a paper in preparation). A 70 µg of aliquot of the MT1-MMP specific antibody was covalently bound to 10 mg of epoxy-functionalized magnetic beads following the provided protocol (Dynabeads M-270 epoxy, Invitrogen). For immunoprecipitation, 1 mL of membrane lysates prepared from wild-type HT1080 cells was incubated 24 h at 4°C with 10 mg of anti-MT1-MMP antibody-conjugated beads. After incubation, the resin was collected with a magnet and washed with lysis buffer. The bound MT1-MMP was eluted with 0.1 M glycine-HCl (pH 3.0), and then neutralized with 1 M Tris-HCl (pH 8.0). The eluate was concentrated to 20 µL (about 7.5 ng protein/µL) by diafiltration using an Amicon Ultra device (cut-off: 10 kDa, Millipore, Billerica, MA).

### FLAG-tagged MT1-MMP Isolation

Isolation of MT1-FLAG was conducted using anti-FLAG M2 magnetic beads (Sigma-Aldrich, St. Louis, Missouri) according to the manufacturer’s protocol. Briefly, 1 mL of membrane lysates prepared from MT1-FLAG revertant HT1080 cells was incubated 16 h at 4°C with 20 µL of the resin. The MT1-FLAG was eluted with 3× FLAG peptide in TBS containing 0.1% CHAPS. The eluate was concentrated to 20 µL (about 25 ng protein/µL) as described above.

### Soluble MT1-MMP Preparation

An MT1-MMP transmembrane deletion mutant was purified from the conditioned media of Madin-Darby canine kidney (MDCK) cells as described previously [26].

### Sialidase Treatment

For desialylation, 500 ng of analyte was treated with 5 mU of sialidase/neuraminidase F (E.C. 3.2.1.18. from *Streptococcus*, Seikagaku Co., Tokyo, Japan) at 37°C for 16 h in 20 µL of TBS containing 0.1% CHAPS.

### SDS-PAGE

Samples in the Laemmli’s sample buffer containing 5% β-mercaptoethanol were boiled for 5 min and then centrifuged at 20,000×g for 5 min at 25°C. The supernatants were subjected to discontinuous Tris−glycine−SDS-PAGE using a 4% stacking gel and a 10% separating gel.

### Western Blot

After electrophoresis, materials in SDS-PAGE gels were electrotransferred onto PVDF membranes (Millipore), and the membranes were blocked with 5% skim milk in PBS. Immunoreactive materials were detected by staining with the indicated antibodies using the chemiluminescence substrate Western Lightning (Perkin Elmer Inc, Waltman, MA). The following antibodies were used in this study: a monoclonal anti-MT1-MMP antibody (222-1D8, a generous gift from Dr. Kazushi Iwata, Daiichi Fine Chemical, Takaoka, Japan), a polyclonal anti-FLAG antibody (Sigma-Aldrich), a monoclonal anti-β-tubulin antibody (E7, Development Studies Hybridoma Bank, University of Iowa, USA), horseradish peroxidase-conjugated anti-mouse IgG antibody and anti-rabbit IgG antibody (GE Healthcare Biosciences, Piscataway, NJ).

### Densitometry

The intensity of Coomassie Blue-stained protein was quantified using ImageJ 1.43 software (National Institutes of Health, Bethesda, MD).

### 3AQ/CHCA Liquid Matrix

Concentrated 3AQ/CHCA was prepared by dissolving 35 mg of 3-aminoquinoline (Fluka Analytical, Sigma-Aldrich) in 150 µL of a saturated solution of α-cyano-4-hydroxycinnamic acid (LaserBio Labs, Sophia-Antipolis Cedex, France) in methanol. The concentrated 3AQ/CHCA solution was diluted 10-fold using 100 mM ammonium phosphate, and referred as the liquid matrix 3AQ/CHCA. A saturated CHCA solution was prepared by dissolving 10 mg of CHCA in 540 µL of 50% acetonitrile/0.1% trifluoroacetic acid and 60 µL of 100 mM ammonium phosphate.

### DHB Matrix

DHB (2,5-dihydroxybenzoic acid) matrix solution was prepared by dissolving 10 mg of DHB (LaserBio Labs) in 1 mL of 50% acetonitrile/0.1% trifluoroacetic acid. For DHB matrix analysis, a 0.5 µL aliquot of tryptic MT1-MMP digest that had been mixed with an equal volume of DHB matrix solution was spotted onto a target plate and allowed to dry.

### Tryptic In-gel Digestion

In-gel digestion was carried out according to the method of Shevchenko *et al* [27]. A region of the gel containing MT1-MMP protein stained with colloidal Coomassie Blue G-250 (SimpleBlue SafeStain, Invitrogen) was excised and cut into small pieces. The MT1-MMP gel pieces were destained with 50 mM ammonium bicarbonate in 50% methanol and then dried under vacuum centrifugation. The dried gel pieces were rehydrated with 10 µL of 1 pmol/µL trypsin (Promega, Madison, WI) in 25 mM ammonium bicarbonate for 30 min at 4°C, to which was added 20 µL of 25 mM ammonium bicarbonate for incubation 16 h at 37°C. The supernatant is referred to as the tryptic MT1-MMP digest.

### Mass Spectrometric Measurements

A 0.5 µL aliquot of tryptic MT1-MMP digest was directly spotted onto the liquid matrix 3AQ/CHCA (0.5 µL) that was mounted on a MALDI target plate before sample application, and incubated for 1 h at 25°C. MALDI-MS was performed using a digital ion trap-type (MALDI-DIT XT) and a 3-D quadrupole ion trap-type (AXIMA-*Resonance*) mass spectrometer (Shimadzu/Kratos, Manchester, U.K.), equipped with a nitrogen UV laser (377 nm wavelength). Argon gas was used for collision-induced dissociation fragmentation, and helium gas was used for ion cooling. MS and MS^n^ were carried out in the positive ion extraction modes. All analyses were performed in a high vacuum condition of 5×10^−5^ Pa or less. Mass spectra were obtained by accumulation of 100 laser shots for each analysis. External mass calibration was carried out using standard peptides.

## Supporting Information

Figure S1 **Preparation of FLAG-tagged MT1-MMP.** (A) MT1-FLAG was expressed in human fibrosarcoma HT1080 cells (Rev) in which the expression of endogenous MT1-MMP was first depleted by using an shRNA targeting its mRNA (KD). Wild-type (WT), KD and Rev HT1080 cells were lysed by boiling in Laemmli’s sample buffer. The level of wild-type and FLAG-tagged MT1-MMP in whole cell lysates was analyzed by Western blot using anti-MT1-MMP antibody (upper panel) and anti-FLAG antibody (middle panel), respectively. MT1-FLAG was expressed at a level similar to that of the endogenous wild-type protein. Tubulin was used as the control for loading of protein extract (lower panel). (B) MT1-FLAG was immunoprecipitated using anti-FLAG affinity resin from membrane lysates of MT1-FLAG revertant cells and was further isolated by SDS-PAGE and staining with Coomassie Blue. A polypeptide corresponding to MT1-FLAG was excised, digested in-gel with trypsin, and analyzed by MS^n^ using the liquid matrix 3AQ/CHCA. Sialidase contains BSA as a carrier protein. Numbers on the left of the panels represent molecular masses in kilodaltons (kDa).
(TIF)

Figure S2 **MS^n^ profile of the ion at** ***m/z*** **1,968.7 derived from the MS spectrum of tryptic MT1-MMP digests.** The ion peak derived from the MS spectrum of tryptic MT1-FLAG digests at *m/z* 1,968.7 (Fig. 3) was subjected to MS^2^ analysis (A) and the product peak at *m/z* 1,024.0 was further analyzed by MS^3^ (B).
(TIF)

Figure S3 **Comparison of MS spectra of tryptic MT1-MMP digests in different matrices.** MS spectrum of tryptic MT1-FLAG digests was obtained within the central area of the liquid matrix 3AQ/CHCA (A), or within the sweet spot of the solid matrix DHB (B). Numbers on the left of the panels represent the cumulative intensity of the top peaks (arbitrary units). The peaks indicated with arrowheads are glycopeptide ions (refer Fig. 3).
(TIF)

Figure S4 **MS spectrum of tryptic MT1-MMP digests derived from MDCK cells.** An aliquot of tryptic MT1-MMP digest derived from MDCK cells was applied directly onto the liquid matrix 3AQ/CHCA on the MALDI target plate and analyzed by MS^n^. MS spectrum was obtained within the central area of the liquid matrix. The glycan moieties and the amino acid sequences of peptides including glycosylation sites of these glycopeptides were assigned by MS^2^ and MS^3^ (data not shown).
(TIF)

Figure S5 **MS spectrum of tryptic MT1-MMP digests within the periphery of 3AQ/CHCA.** An aliquot of tryptic MT1-FLAG digest was applied directly onto the liquid matrix 3AQ/CHCA on the MALDI target plate. MS spectrum was obtained within the periphery of the liquid matrix (closed circle [•]). A stereoscopic microscope image of the sample spot is shown in the left upper insert. Nonglycosylated peptides derived from MT1-FLAG digests are indicated by star (*). The amino acid sequences of the peptides were confirmed by MS^2^ (data not shown).
(TIF)

Figure S6 **Sequence coverage of MT1-MMP by MS measurements using the liquid matrix 3AQ/CHCA.** Peptides derived from tryptic MT1-FLAG digests identified in the center and in the periphery of the liquid matrix 3AQ/CHCA by MS analysis are labeled in yellow and green, respectively. Overall, approximately 50% sequence coverage of the extracellular region of MT1-MMP was achieved.
(TIF)
