# Supplementary figures and images for: Detection of the Heterogeneous O-Glycosylation Profile of MT1-MMP Expressed in Cancer Cells by a Simple MALDI-MS Method
Source: PLoS One. 2012 Aug 22;7(8):e43751. doi: 10.1371/journal.pone.0043751 (PMC3425508; doi:10.1371/journal.pone.0043751)

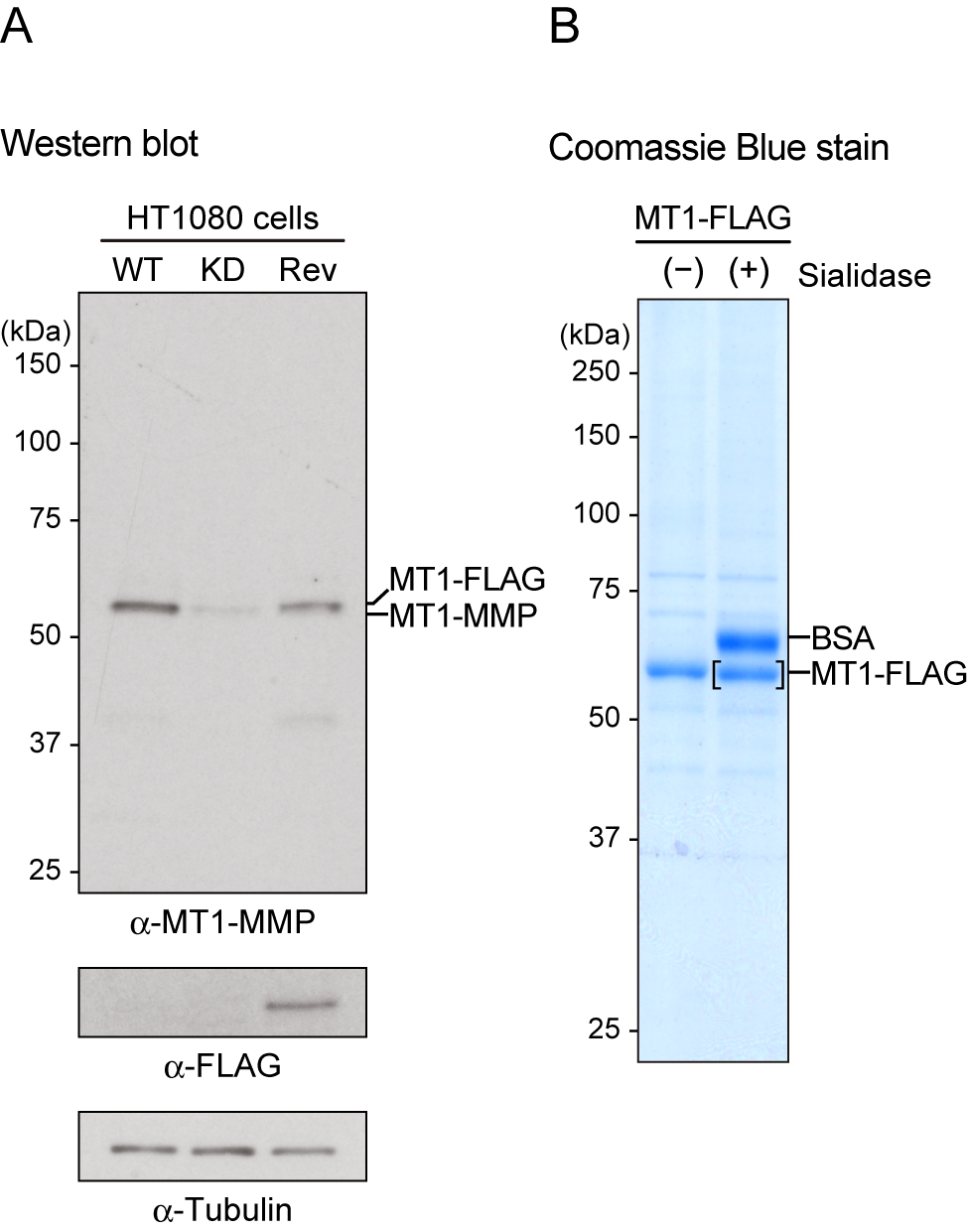

Supplement: Figure S1 — Preparation of FLAG-tagged MT1-MMP. (A) MT1-FLAG was expressed in human fibrosarcoma HT1080 cells (Rev) in which the expression of endogenous MT1-MMP was first depleted by using an shRNA targeting its mRNA (KD). Wild-type (WT), KD and Rev HT1080 cells were lysed by boiling in Laemmli’s sample buffer. The level of wild-type and FLAG-tagged MT1-MMP in whole cell lysates was analyzed by Western blot using anti-MT1-MMP antibody (upper panel) and anti-FLAG antibody (middle panel), respectively. MT1-FLAG was expressed at a level similar to that of the endogenous wild-type protein. Tubulin was used as the control for loading of protein extract (lower panel). (B) MT1-FLAG was immunoprecipitated using anti-FLAG affinity resin from membrane lysates of MT1-FLAG revertant cells and was further isolated by SDS-PAGE and staining with Coomassie Blue. A polypeptide corresponding to MT1-FLAG was excised, digested in-gel with trypsin, and analyzed by MSn using the liquid matrix 3AQ/CHCA. Sialidase contains BSA as a carrier protein. Numbers on the left of the panels represent molecular masses in kilodaltons (kDa). (TIF) [file pone.0043751.s001.tif]

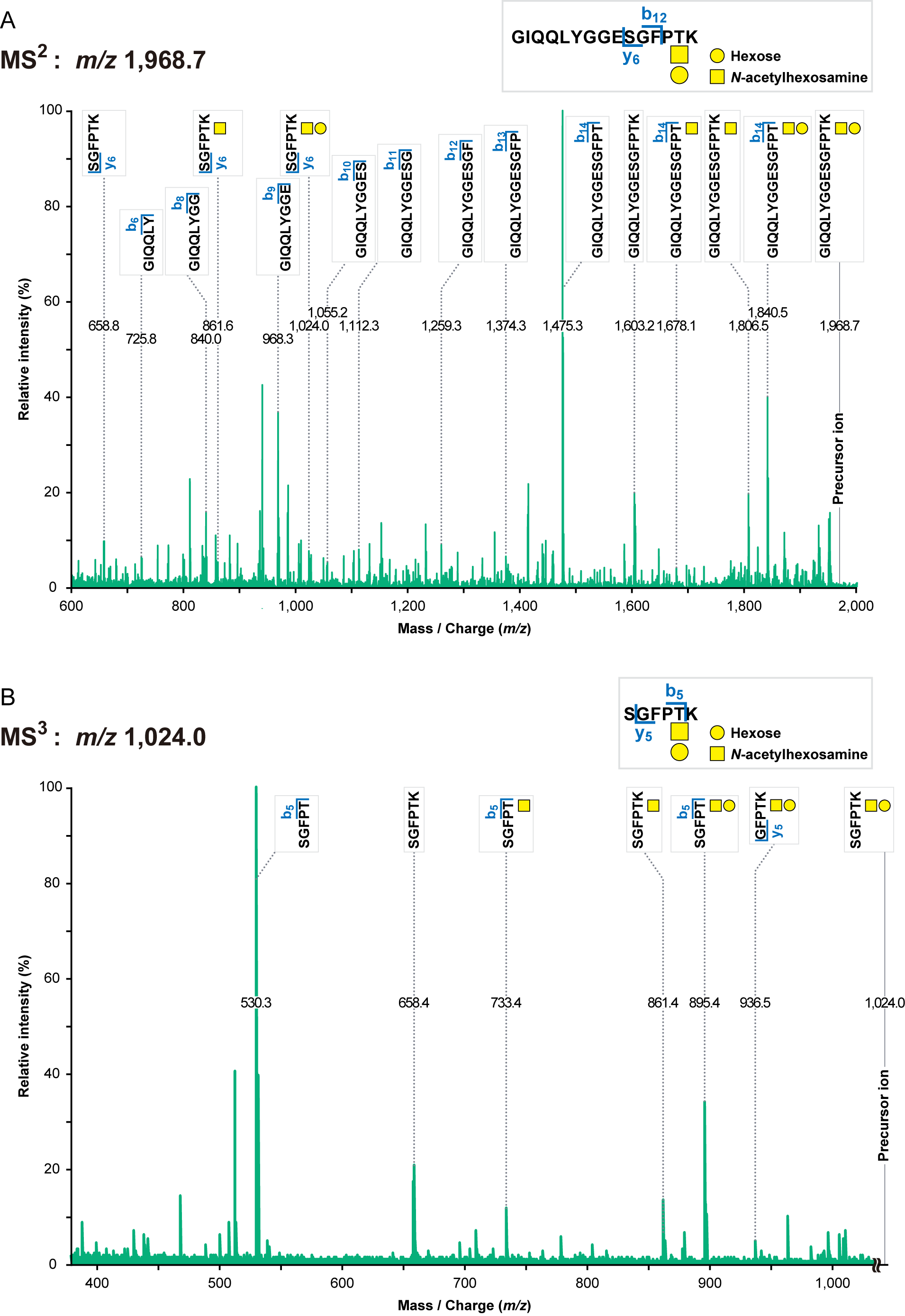

Supplement: Figure S2 — MSn profile of the ion at m/z 1,968.7 derived from the MS spectrum of tryptic MT1-MMP digests. The ion peak derived from the MS spectrum of tryptic MT1-FLAG digests at m/z 1,968.7 (Fig. 3) was subjected to MS2 analysis (A) and the product peak at m/z 1,024.0 was further analyzed by MS3 (B). (TIF) [file pone.0043751.s002.tif]

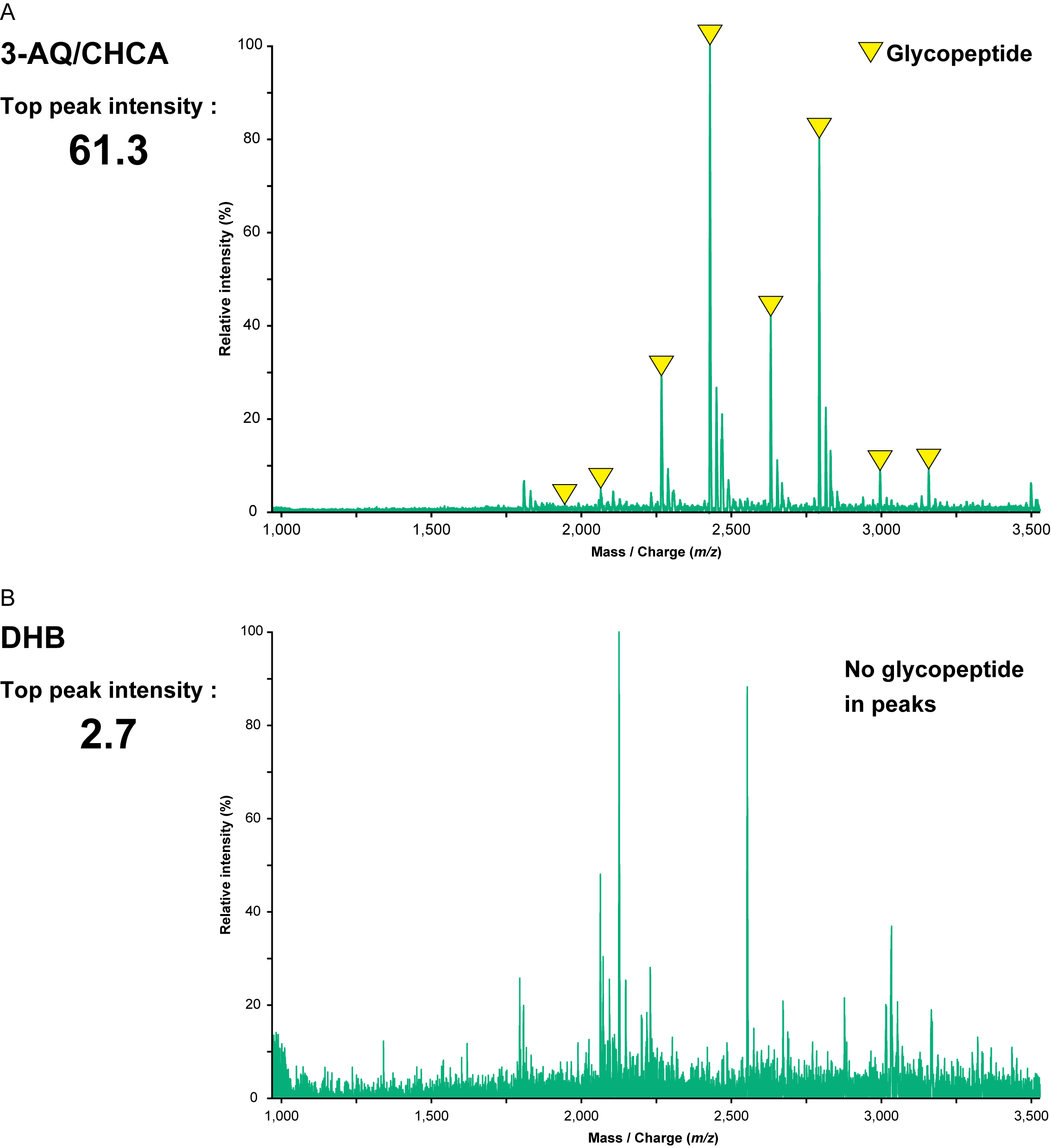

Supplement: Figure S3 — Comparison of MS spectra of tryptic MT1-MMP digests in different matrices. MS spectrum of tryptic MT1-FLAG digests was obtained within the central area of the liquid matrix 3AQ/CHCA (A), or within the sweet spot of the solid matrix DHB (B). Numbers on the left of the panels represent the cumulative intensity of the top peaks (arbitrary units). The peaks indicated with arrowheads are glycopeptide ions (refer Fig. 3). (TIF) [file pone.0043751.s003.tif]

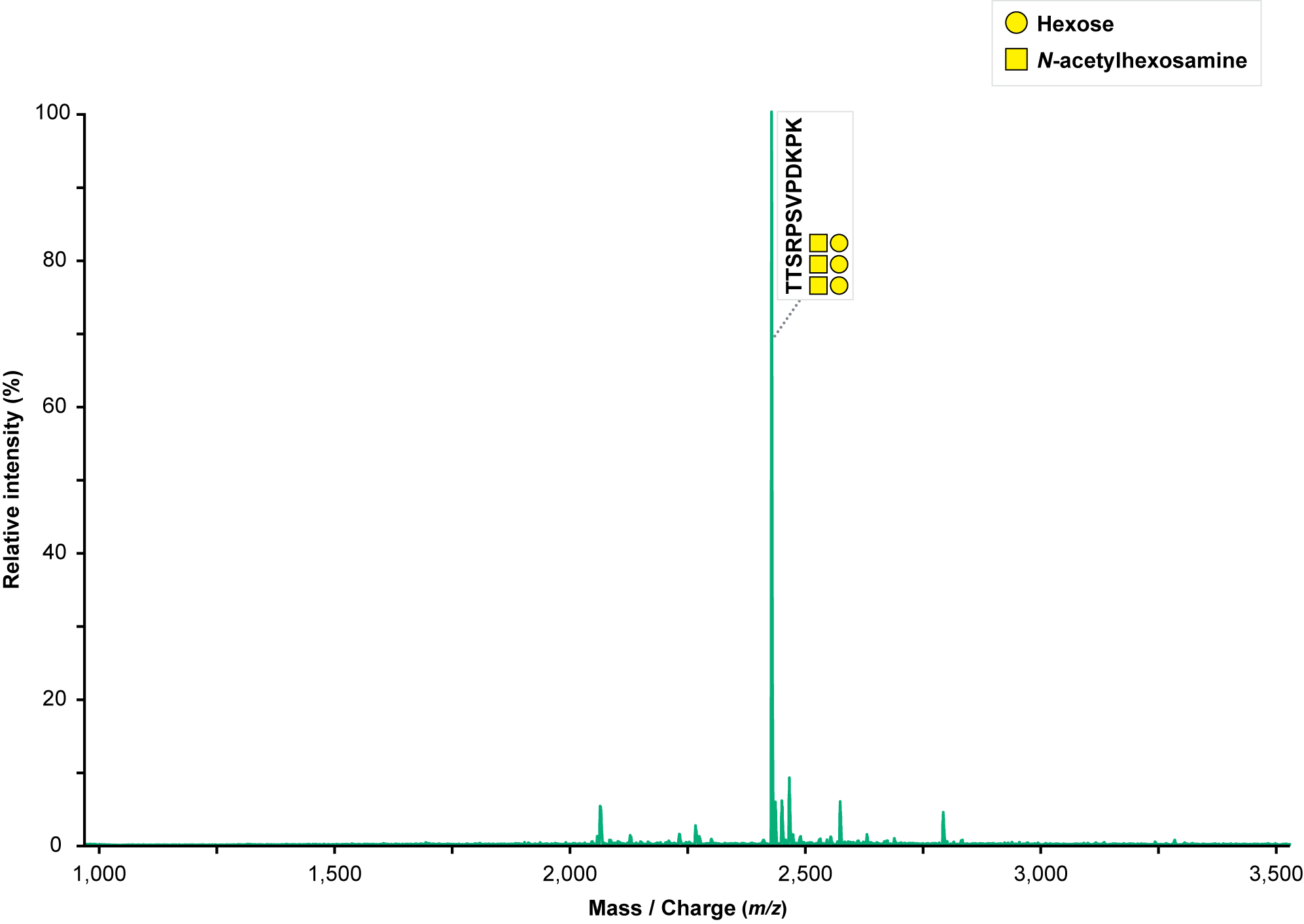

Supplement: Figure S4 — MS spectrum of tryptic MT1-MMP digests derived from MDCK cells. An aliquot of tryptic MT1-MMP digest derived from MDCK cells was applied directly onto the liquid matrix 3AQ/CHCA on the MALDI target plate and analyzed by MSn. MS spectrum was obtained within the central area of the liquid matrix. The glycan moieties and the amino acid sequences of peptides including glycosylation sites of these glycopeptides were assigned by MS2 and MS3 (data not shown). (TIF) [file pone.0043751.s004.tif]

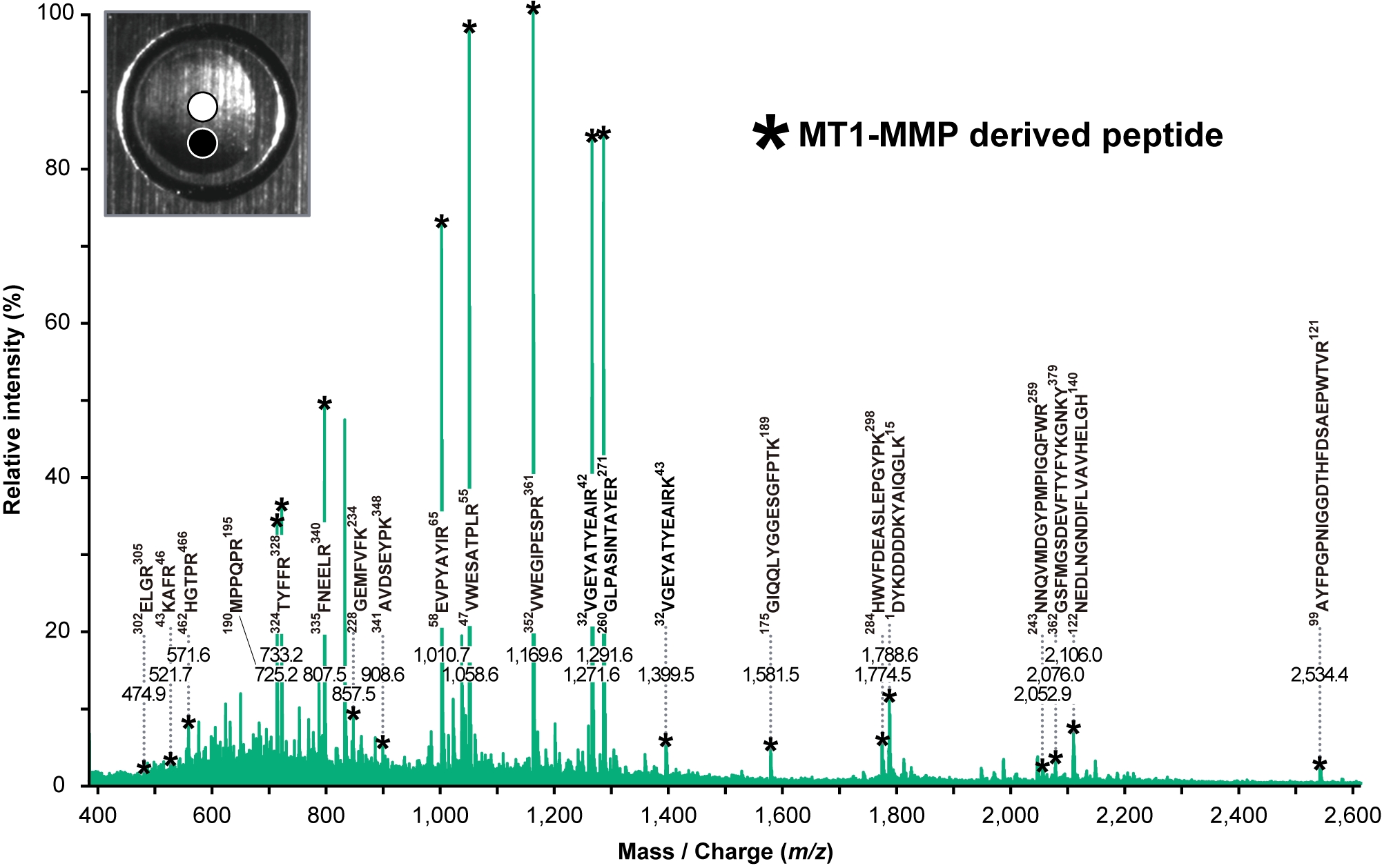

Supplement: Figure S5 — MS spectrum of tryptic MT1-MMP digests within the periphery of 3AQ/CHCA. An aliquot of tryptic MT1-FLAG digest was applied directly onto the liquid matrix 3AQ/CHCA on the MALDI target plate. MS spectrum was obtained within the periphery of the liquid matrix (closed circle [•]). A stereoscopic microscope image of the sample spot is shown in the left upper insert. Nonglycosylated peptides derived from MT1-FLAG digests are indicated by star (*). The amino acid sequences of the peptides were confirmed by MS2 (data not shown). (TIF) [file pone.0043751.s005.tif]

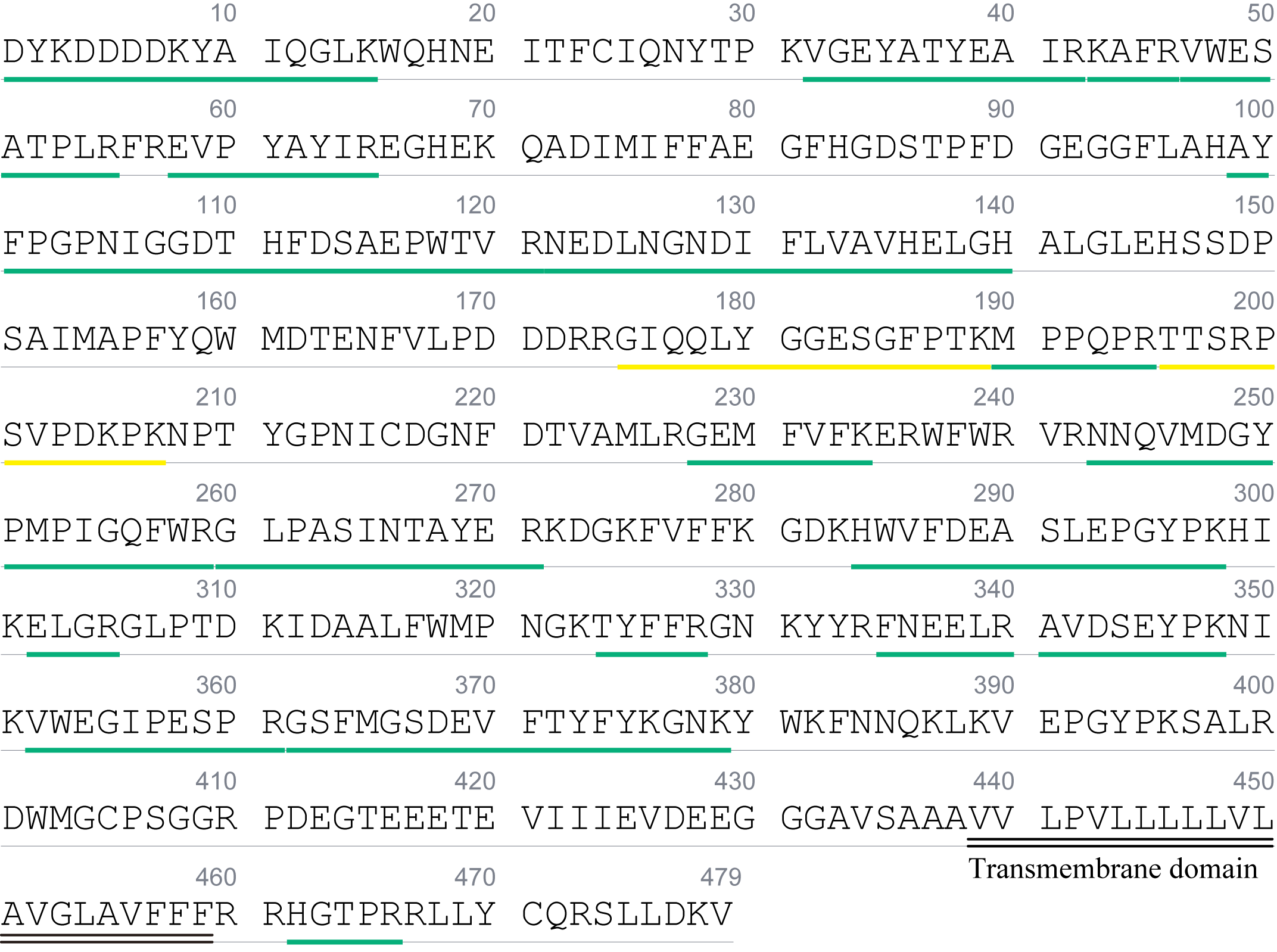

Supplement: Figure S6 — Sequence coverage of MT1-MMP by MS measurements using the liquid matrix 3AQ/CHCA. Peptides derived from tryptic MT1-FLAG digests identified in the center and in the periphery of the liquid matrix 3AQ/CHCA by MS analysis are labeled in yellow and green, respectively. Overall, approximately 50% sequence coverage of the extracellular region of MT1-MMP was achieved. (TIF) [file pone.0043751.s006.tif]
